# Supplementary material for: Air pollution-induced proteomic alterations increase the risk of child respiratory infections
Source: Nat Commun. 2025 Jul 1;16:5930. doi: 10.1038/s41467-025-61392-y (PMC12217977; doi:10.1038/s41467-025-61392-y)

Supplementary Information

Supplementary Table 1
Overview of proteins in the maternal inflammatory panel.

**No.**	**Short name**	**Full name**	**Classification**	**Limit of detection (LOD)**	**Data frequency below LOD**
1	4EBP1	Eukaryotic translation initiation factor 4E-binding protein 1	translation factor	0,30582	0,0%
2	ADA	Adenosine deaminase	deaminase	0,16755	0,0%
3	ARTN	Artemin	neurotrophic factor	-0,68723	63,4%
4	AXIN1	Axin-1	G-protein modulator	0,3282	3,1%
5	BDNF	Brain-derived neurotrophic factor	neurotrophic factor	1,64851	100,0%
6	BetaNGF	Beta-nerve growth factor	neurotrophic factor	-0,06059	2,3%
7	CASP8	Caspase-8	cysteine protease	0,08481	0,0%
8	CCL11	Eotaxin	chemokine	-0,58412	0,0%
9	CCL13/MCP4	C-C motif chemokine 13/Monocyte chemotactic protein 4	chemokine	0,75934	0,0%
10	CCL19	C-C motif chemokine 19	chemokine	0,73072	0,0%
11	CCL2/MCP1	C-C motif chemokine 2/Monocyte chemotactic protein 1	chemokine	-0,31721	0,0%
12	CCL20	C-C motif chemokine 20	chemokine	-0,18401	0,0%
13	CCL23	C-C motif chemokine 23	chemokine	0,24031	0,0%
14	CCL25	C-C motif chemokine 25	chemokine	0,54183	0,0%
15	CCL28	C-C motif chemokine 28	chemokine	-0,47753	0,0%
16	CCL3/MIP1alpha	C-C motif chemokine 3	chemokine	-0,56203	0,0%
17	CCL4	C-C motif chemokine 4	chemokine	-0,39105	0,0%
18	CCL7/MCP3	C-C motif chemokine 7/Monocyte chemotactic protein 3	chemokine	-0,69578	0,0%
19	CCL8/MCP2	C-C motif chemokine 8/Monocyte chemotactic protein 2	chemokine	0,26212	0,0%
20	CD244	Natural killer cell receptor 2B4	cell adhesion molecule	-0,342	0,0%
21	CD40	Tumor necrosis factor receptor superfamily member 5	tumor necrosis factor receptor	-0,40036	0,0%
22	CD5	T-cell surface glycoprotein CD5	oxidase	1,55682	0,0%
23	CD6	T-cell differentiation antigen CD6	oxidase	0,24708	0,0%
24	CDCP1	CUB domain-containing protein 1	transmembrane glycoprotein	0,23778	0,0%
25	CSF1	Macrophage colony-stimulating factor 1	cytokine	0,21516	0,0%
26	CST5	Cystatin-D	cysteine protease inhibitor	0,46149	0,0%
27	CX3CL1	Fractalkine	chemokine	0,27471	0,0%

28	CXCL1	Growth-regulated alpha protein	chemokine	-0,64624	0,0%
29	CXCL10	C-X-C motif chemokine 10	chemokine	-0,49106	0,0%
30	CXCL11	C-X-C motif chemokine 11	chemokine	-0,68731	0,0%
31	CXCL5	C-X-C motif chemokine 5	chemokine	-0,61454	0,0%
32	CXCL6	C-X-C motif chemokine 6	chemokine	0,63598	0,0%
33	CXCL9	C-X-C motif chemokine 9	chemokine	-0,12245	1,2%
34	DNER	Delta and Notch-like epidermal growth factor-related receptor	growth factor	-0,8628	0,0%
35	EN-RAGE/S100A12	Protein S100-A12	calmodulin	0,20079	43,0%
36	FGF19	Fibroblast growth factor 19	growth factor	1,16716	0,0%
37	FGF21	Fibroblast growth factor 21	growth factor	-0,41984	5,3%
38	FGF23	Fibroblast growth factor 23	growth factor	0,08019	0,0%
39	FGF5	Fibroblast growth factor 5	growth factor	1,73459	0,0%
40	FLT3L	Fms-related tyrosine kinase 3 ligand	cytokine	-0,03432	97,1%
41	GDNF	Glial cell line-derived neurotrophic factor	neurotrophic factor	-0,26237	47,0%
42	HGF	Hepatocyte growth factor	growth factor	0,29265	0,0%
43	IFNgamma	Interferon gamma	interferon superfamily	-1,12684	0,0%
44	IL10	Interleukin-10	interleukin superfamily	-0,12492	17,2%
45	IL10RA	Interleukin-10 receptor subunit alpha	defense/immunity protein	-0,37838	88,3%
46	IL10RB	Interleukin-10 receptor subunit beta	defense/immunity protein	-0,08755	1,7%
47	IL12B	Interleukin-12 subunit beta	interleukin superfamily	-0,43973	0,0%
48	IL13	Interleukin-13	interleukin superfamily	0,01482	93,5%
49	IL15RA	Interleukin-15 receptor subunit alpha	cytokine receptor	-0,58126	51,8%
50	IL17A	Interleukin-17A	interleukin superfamily	0,39295	91,7%
51	IL17C	Interleukin-17C	chemokine	1,05127	92,3%
52	IL18	Interleukin-18	interleukin superfamily	-0,31023	35,0%
53	IL18R1	Interleukin-18 receptor 1	type I cytokine receptor	0,44917	2,8%
54	IL1alpha	Interleukin-1 alpha	interleukin superfamily	-0,5365	90,0%
55	IL2	Interleukin-2	interleukin superfamily	0,31825	0,0%
56	IL20	Interleukin-20	interleukin superfamily	0,08212	99,9%
57	IL20RA	Interleukin-20 receptor subunit alpha	defense/immunity protein	0,58866	98,3%
58	IL22RA1	Interleukin-22 receptor subunit alpha-1	defense/immunity protein	-0,48356	78,6%
59	IL24	Interleukin-24	interleukin superfamily	-0,39696	72,0%
60	IL2RB	Interleukin-2 receptor subunit beta	type I cytokine receptor	-0,26766	0,0%

61	IL33	Interleukin-33	interleukin superfamily	-0,41025	12,6%
62	IL4	Interleukin-4	interleukin superfamily	0,18175	0,0%
63	IL5	Interleukin-5	interleukin superfamily	0,19345	0,0%
64	IL6	Interleukin-6	interleukin superfamily	-0,56224	91,3%
65	IL7	Interleukin-7	interleukin superfamily	0,23421	0,0%
66	IL8/CXCL8	Interleukin-8	chemokine	0,02541	0,0%
67	KITLG/SCF	Kit ligand/Stem cell factor	cell adhesion molecule	0,05476	0,0%
68	LIF	Leukemia inhibitory factor	cytokine	-0,14162	66,9%
69	LIFR	Leukemia inhibitory factor receptor	cytokine	2,1855	0,0%
70	LTA/TNFB	Lymphotoxin-alpha/TNF-beta	tumor necrosis factor family member	0,25021	0,0%
71	MMP1	Interstitial collagenase	extracellular matrix organization	0,07358	0,0%
72	MMP10	Stromelysin-2	extracellular matrix organization	-0,3642	87,3%
73	NRTN	Neurturin	neurotrophic factor	0,41879	0,1%
74	NTF3/NT3	Neurotrophin-3	neurotrophic factor	-0,05434	0,0%
75	OSM	Oncostatin-M	interleukin superfamily	-0,21844	0,0%
76	PDL1	Programmed cell death 1 ligand 1	immunoglobulin receptor superfamily	0,85911	0,0%
77	PLAU/uPA	Urokinase-type plasminogen activator	serine protease	-0,27057	0,0%
78	SIRT2	NAD-dependent protein deacetylase sirtuin-2	chromatin/chromatin-binding protein	-0,27665	0,5%
79	SLAMF1	Signaling lymphocytic activation molecule	cell adhesion molecule	-0,09034	10,9%
80	STAMBP	STAM-binding protein	cytokine	0,01604	8,8%
81	SULT1A1/ST1A1	Sulfotransferase 1A1	transferase	0,21967	0,0%
82	TGFalpha	Transforming growth factor alpha	growth factor	-0,85988	0,0%
83	TGFB1/LAP-TGFbeta1	Latency-associated peptide Transforming growth factor beta-1	growth factor	-0,44285	0,0%
84	TNF	Tumor necrosis factor	tumor necrosis factor family member	-0,16998	0,0%
85	TNFRSF11B/OPG	Tumor necrosis factor receptor superfamily member 11B/Osteoprotegerin	tumor necrosis factor receptor	-0,55347	0,0%
86	TNFRSF9	Tumor necrosis factor receptor superfamily member 9	tumor necrosis factor receptor	0,30532	0,0%
87	TNFSF10/TRAIL	Tumor necrosis factor ligand superfamily member 10/TNF-related apoptosis-inducing ligand	tumor necrosis factor family member	-0,24974	0,0%
88	TNFSF11/TRANCE	Tumor necrosis factor ligand superfamily member 11	tumor necrosis factor family member	0,11356	0,0%

89	TNFSF12/TWEAK	Tumor necrosis factor ligand superfamily member 12	tumor necrosis factor family member	0,22551	94,1%
90	TNFSF14	Tumor necrosis factor ligand superfamily member 14	tumor necrosis factor family member	-0,39638	0,0%
91	TSLP	Thymic stromal lymphopoietin	cytokine	-0,00747	0,0%
92	VEGFA	Vascular endothelial growth factor A	growth factor	1,66597	0,0%

Supplementary Table 2

The mean number of infection episodes during the first 3 years of life in $COPSAC_{2010}$ from daily diaries.

**Type of infection**	**Mean (SD)**
Total number of infections	16.4 (8.3)
Cold	12.2 (8.0)
Pneumonia	0.60 (1.09)
Tonsillitis	0.38 (0.93)
Gastric infections	1.74 (1.78)
Acute otitis media	1.63 (2.12)
Fever	5.60 (4.01)

Supplementary Table 3

Association between air pollution exposure from birth to age 1 year and risk of respiratory infections age 1-2 years in the EMIL cohort.

**Air pollution exposure**	**Estimate**	**95% CI**	**P value**
$PM_{2.5}$	0.62	0.24-1.60	0.32
PM_{10}	0.90	0.71-1.15	0.40
NO_2	0.99	0.90-1.09	0.79

Supplementary Figure 1

Heatmap. Heatmap of associations between the 92 inflammatory proteins.

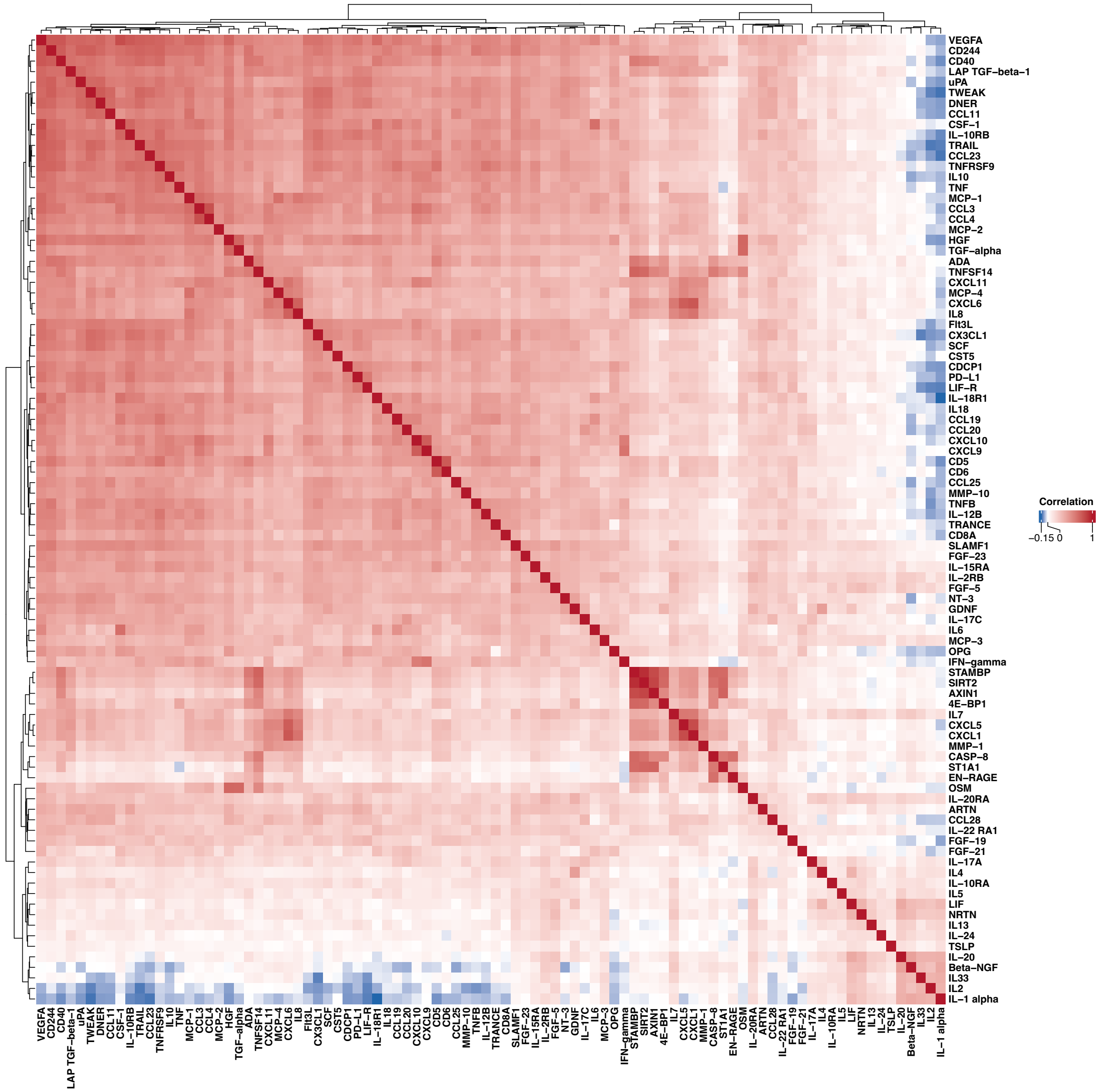

Supplementary Figure 2

Flowchart. Flowchart of the participants in $COPSAC_{2010}$

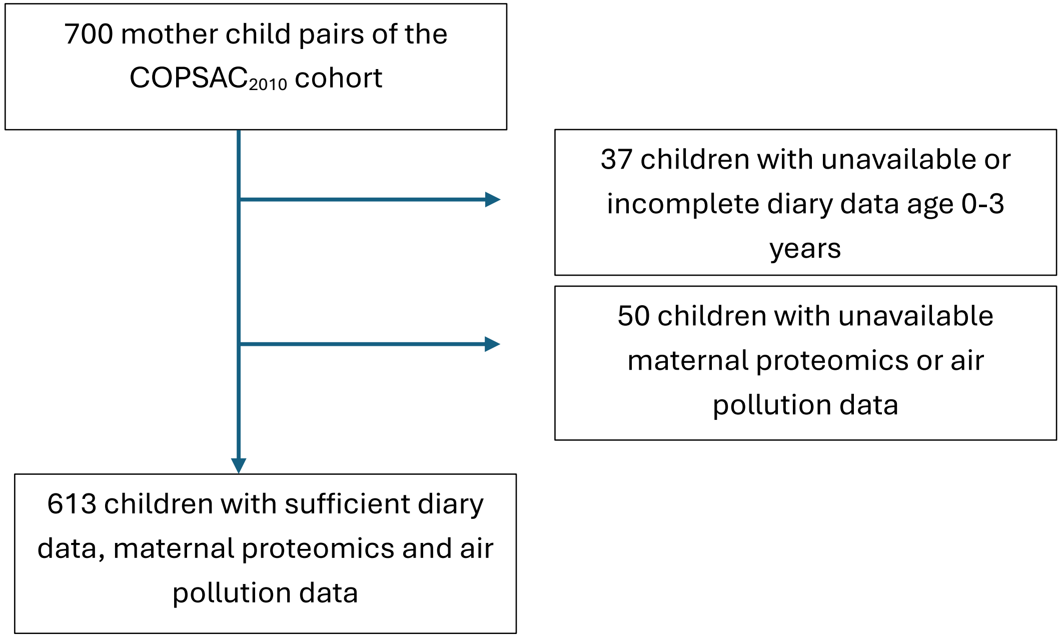

Supplementary Figure 3

Prenatal air pollution vs infections in COPSAC$_{2010}$. Association analyses between prenatal air pollution exposure and infection types in COPSAC$_{2010}$. A) PM2.5 exposure, B) PM10 exposure and C) NO_2 exposure. Estimates derived from Quasi-Poisson regression models with 95% confidence intervals adjusted for gestational age, furred pets during the first year, maternal education and income, time to daycare start, number of older siblings, alcohol use, antibiotic use and smoking during pregnancy, delivery mode, child hospitalization at birth and birth season (n=613).

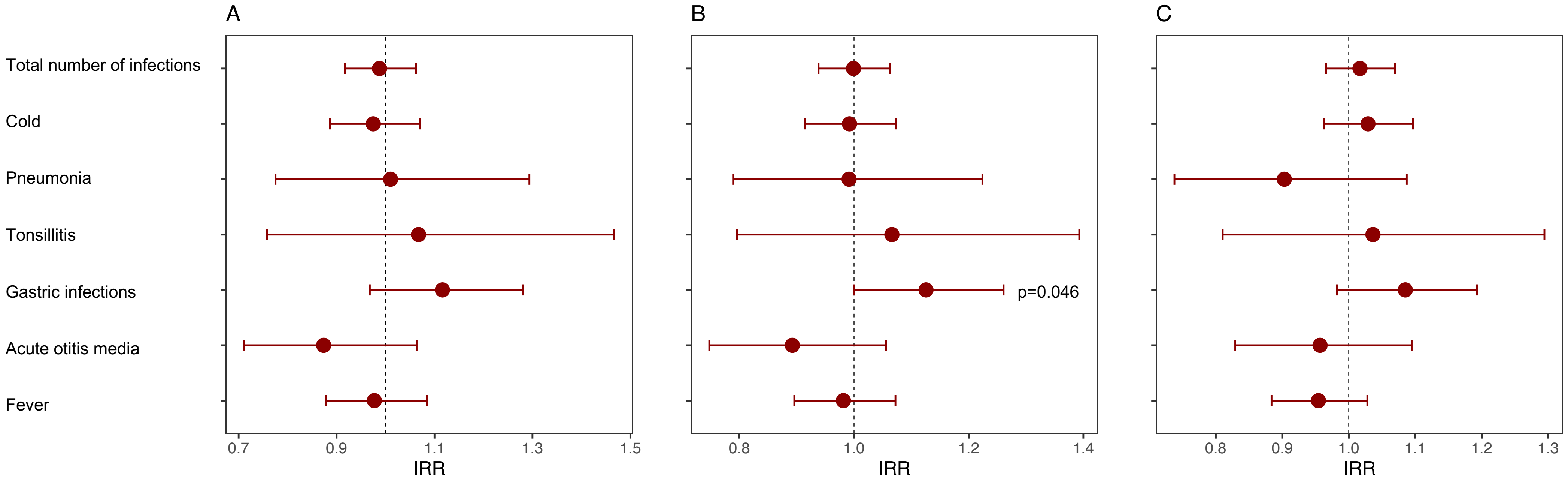

Supplementary Figure 4

Volcano plots of proteins in COPSAC$_{2010}$. Volcano plots of the associations between $PM_{2.5}$, PM_{10} and NO_2 and maternal inflammatory proteomic profile in the COPSAC$_{2010}$ cohort. Dotted line represents FDR adjusted p-values.

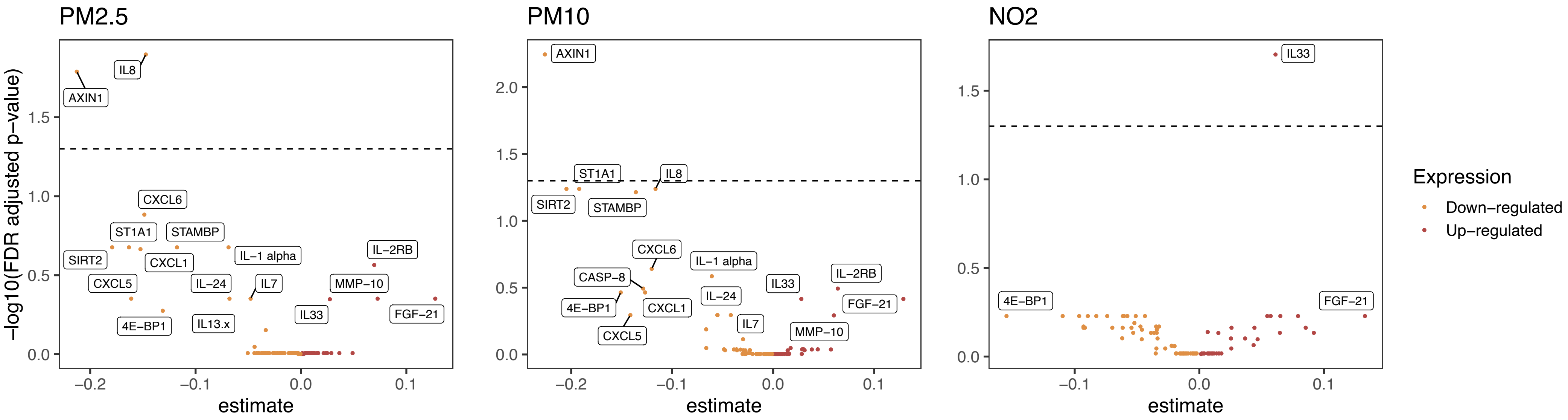

Supplementary Figure 5

Principal component 2 vs risk of infections in COPSAC$_{2010}$. Associations between principal component 2 and risk of infections age 0-3 years in the COPSAC$_{2010}$. Estimates derived from Quasi-Poisson regression models with 95% confidence intervals adjusted for gestational age, furred pets during the first year, maternal education and income, time to daycare start, number of older siblings, alcohol use, antibiotic use and smoking during pregnancy, delivery mode, child hospitalization at birth and birth season (n=613).

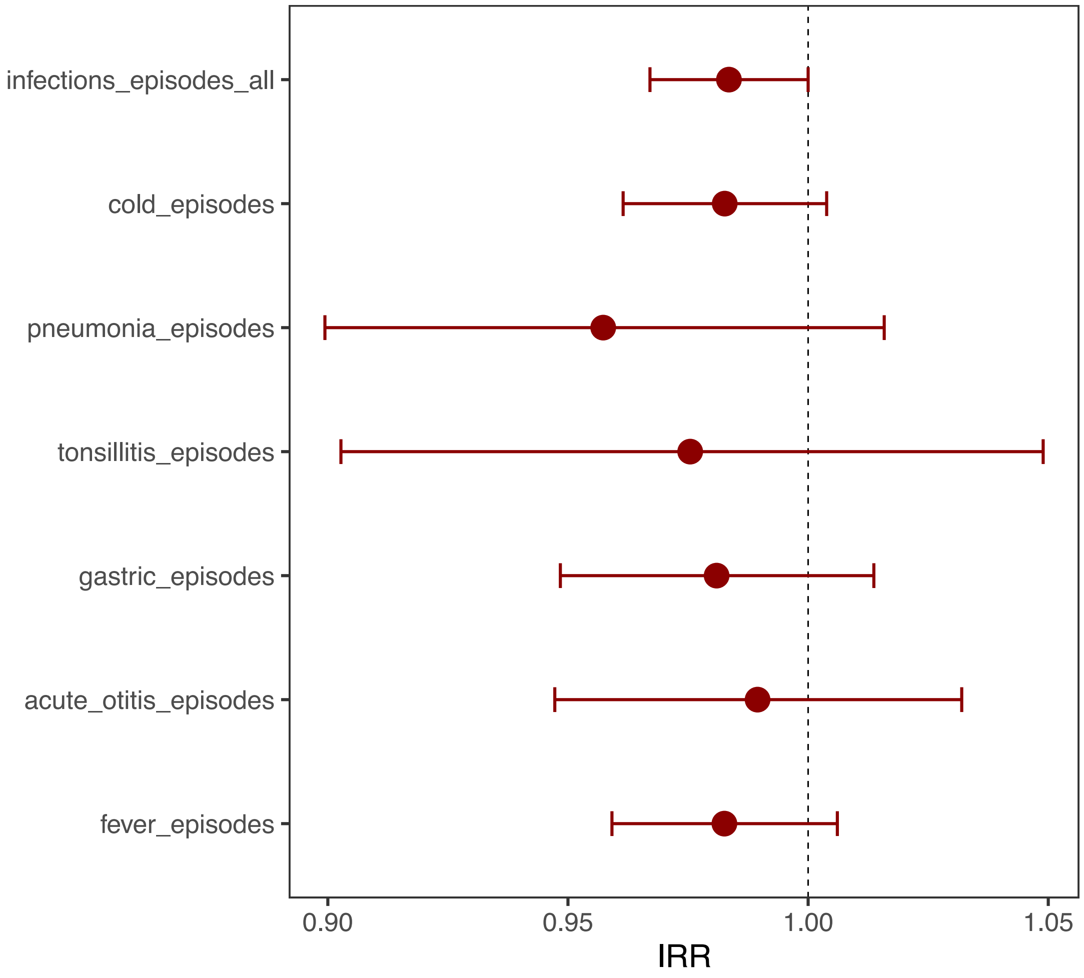

Supplement: Supplementary file 1 — Supplementary Information [file 41467_2025_61392_MOESM1_ESM.pdf]
