# Supplementary material for: Air pollution-induced proteomic alterations increase the risk of child respiratory infections
Source: Nat Commun. 2025 Jul 1;16:5930. doi: 10.1038/s41467-025-61392-y (PMC12217977; doi:10.1038/s41467-025-61392-y)
Supplement: Supplementary file 2 — Reporting Summary [file 41467_2025_61392_MOESM2_ESM.pdf]

nature portfolio

Corresponding author(s): Nicklas Brustad

Last updated by author(s): Apr 26, 2025

Reporting Summary

Nature Portfolio wishes to improve the reproducibility of the work that we publish. This form provides structure for consistency and transparency in reporting. For further information on Nature Portfolio policies, see our Editorial Policies and the Editorial Policy Checklist.

Statistics

For all statistical analyses, confirm that the following items are present in the figure legend, table legend, main text, or Methods section.

n/a	Confirmed	
☐	☒	The exact sample size (*n*) for each experimental group/condition, given as a discrete number and unit of measurement
☐	☒	A statement on whether measurements were taken from distinct samples or whether the same sample was measured repeatedly
☐	☒	The statistical test(s) used AND whether they are one- or two-sided<br>*Only common tests should be described solely by name; describe more complex techniques in the Methods section.*
☐	☒	A description of all covariates tested
☐	☒	A description of any assumptions or corrections, such as tests of normality and adjustment for multiple comparisons
☐	☒	A full description of the statistical parameters including central tendency (e.g. means) or other basic estimates (e.g. regression coefficient) AND variation (e.g. standard deviation) or associated estimates of uncertainty (e.g. confidence intervals)
☐	☒	For null hypothesis testing, the test statistic (e.g. *F*, *t*, *r*) with confidence intervals, effect sizes, degrees of freedom and *P* value noted<br>*Give P values as exact values whenever suitable.*
☒	☐	For Bayesian analysis, information on the choice of priors and Markov chain Monte Carlo settings
☒	☐	For hierarchical and complex designs, identification of the appropriate level for tests and full reporting of outcomes
☒	☐	Estimates of effect sizes (e.g. Cohen's *d*, Pearson's *r*), indicating how they were calculated

Our web collection on statistics for biologists contains articles on many of the points above.

Software and code

Policy information about availability of computer code

Data collection	NA
Data analysis	All analyses were performed with R software (v4.0.3; R Foundation for Statistical Computing, Vienna, Austria).<br>The custom code employed in this research is freely accessible to the public for transparency and reproducibility purposes at https://github.com/tiwa1125/Air-pollution-induced-proteomic-alterations-increase-the-risk-of-child-respiratory-infections.

For manuscripts utilizing custom algorithms or software that are central to the research but not yet described in published literature, software must be made available to editors and reviewers. We strongly encourage code deposition in a community repository (e.g. GitHub). See the Nature Portfolio guidelines for submitting code & software for further information.

Data

Policy information about availability of data

All manuscripts must include a data availability statement. This statement should provide the following information, where applicable:

- Accession codes, unique identifiers, or web links for publicly available datasets
- A description of any restrictions on data availability
- For clinical datasets or third party data, please ensure that the statement adheres to our policy

For the COPSAC2010 cohort dataset, participant-level personally identifiable data are protected under the Danish Data Protection Act and European Regulation 2016/679 of the European Parliament and of the Council that prohibit distribution even in pseudo-anonymised form. However, participant-level data can be made

available under a data-transfer agreement as part of a collaboration effort with COPSAC. Deidentified participant data will be available upon request to the corresponding author with publication to researchers whose proposed use of the data has been approved with a signed data access statement.
For EMIL - participant-level personally identifiable data are protected under the Swedish Data Protection Act (2018:218) and European Regulation 2016/679 of the European Parliament and of the Council hat prohibit distribution even in pseudo-anonymised form. Deidentified participant data will be available upon request

Research involving human participants, their data, or biological material

Policy information about studies with human participants or human data. See also policy information about sex, gender (identity/presentation), and sexual orientation and race, ethnicity and racism.

Reporting on sex and gender	No sex or gender based analyses were performed
Reporting on race, ethnicity, or other socially relevant groupings	No race, ethnicity or other socially relevent grouping based analyses were performed
Population characteristics	Pregnant women and their offspring aged 0-3 years from a Danish population based cohort. Children aged 0-2 years from the Swedish population based EMIL cohort.
Recruitment	Pregnant women around Copenhagen were recruited during their first pregnancy visit (COPSAC). Parents of children born between 2014-2017 in Stockholm were contacted for recruitment of their children. For EMIL, parents of children born from 2014-2017 in Stockholm, Sweden, were invited to participate with the purpose of investigating biological mechanisms behind the adverse respiratory health effects of air pollution in children during early life
Ethics oversight	The study was approved by the Danish ethics committee (H-B-2008-093) for COPSAC and the Swedish ethics comittee from the Regional Ethics Review Board, Karolinska Institute, Stockholm, Sweden for EMIL.

Note that full information on the approval of the study protocol must also be provided in the manuscript.

Field-specific reporting

Please select the one below that is the best fit for your research. If you are not sure, read the appropriate sections before making your selection.

☒ Life sciences ☐ Behavioural & social sciences ☐ Ecological, evolutionary & environmental sciences

For a reference copy of the document with all sections, see nature.com/documents/nr-reporting-summary-flat.pdf

Life sciences study design

All studies must disclose on these points even when the disclosure is negative.

Sample size	Sample sizes were based on asthma prevalence and not calculated for this outcome
Data exclusions	Children with no full follow-up with diary data between age 0-3 years were excluded
Replication	NA
Randomization	NA
Blinding	NA

Reporting for specific materials, systems and methods

We require information from authors about some types of materials, experimental systems and methods used in many studies. Here, indicate whether each material, system or method listed is relevant to your study. If you are not sure if a list item applies to your research, read the appropriate section before selecting a response.

Materials & experimental systems

n/a	Involved in the study
☒	☐ Antibodies
☒	☐ Eukaryotic cell lines
☒	☐ Palaeontology and archaeology
☒	☐ Animals and other organisms
☒	☐ Clinical data
☒	☐ Dual use research of concern
☒	☐ Plants

Methods

n/a	Involved in the study
☒	☐ ChIP-seq
☒	☐ Flow cytometry
☒	☐ MRI-based neuroimaging

Plants

Seed stocks	NA
Novel plant genotypes	NA
Authentication	NA
